# The Cinchona Cure for Intemperance

**Published:** 1880-02

**Authors:** Chas. Warrington Earle

**Affiliations:** Professor of Diseases of Children, Woman’s Medical College, and Physician to the Washingtonian Home, Chicago


### ARTICLE III.

THE CINCHONA CURE FOR INTEMPERANCE. By CHAS. WARRINGTON EARLE, M.D., Professor of Diseases of Children, Woman's Medical College, and Physician to the Washingtonian Home, Chicago.

During the past two years those particularly interested in the subject of temperance in the city have had their attention repeatedly directed to an "Unfailing Cure for Drunkenness."

The discoverer says: "I can and do cure any case of drunkenness that comes to me." He also claims that drunkenness is a disease concerning which there should be no dispute, and that the drug cinchona, when pure, will certainly cure not only the disease but the appetite for alcoholics.

A pamphlet has been published, entitled "Dipsomania. Dr. D'Unger's Cinchona Rubra Cure for Drunkenness—Continuous or Periodical. What it is. How it was discovered. What it has done. Copyrighted, 1879, by Dr. D'Unger."

At the top of the cover of this pamphlet occurs this remarkable sentence: "To Physicians, $10 per pint, six concentrations." Concerning these concentrations I shall have something to say later in this paper.

This monograph on dipsomania has undoubtedly been circulated very widely, as in a letter dated June 9th, 1879, Scotland, I received the following clipping or extract:

> "At the Chicago Washingtonian Home, an institution for the restoration of inebriates, four patients of the worst description—and two of whom had been inmates of various reformatory institutions—have been completely cured by the use of my Cinchona Rubra. The patients can be seen personally by parties interested."

The letter was from a lady, who was greatly distressed in regard to a gentleman addicted to intemperance, and in closing, she said: "Do pray give me your unbiased opinion in regard to this alleged cure."

A number of newspapers, among them several devoted particularly to temperance, have commented freely, and, in many cases, favorably upon the cure. The propriety of so-called temperance organs advocating the use of this drug will become apparent when an analysis of the compound is considered.

It was at the time when the Chicago *Tribune* was giving the cinchona its hearty endorsement and various inquiries were being addressed to me, as the physician of the Washingtonian Home, as to the potency of the drug in curing drunkenness; and after three or four men had come under my care who had been prescribed for by Dr. D'Unger, with no apparent benefit, that I published the following in the *Tribune* of this city:

THE CINCHONA CURE.

CHICAGO, December 28, 1878.

*To the Editor of The Tribune:*

I have very grave doubts as to the efficacy of the cure for drunkenness, advertised by Dr. D'Unger. God knows that no man has the interest of the drunkard more deeply at heart than I have, but I have already the history of several cases in which it has been of no use whatever, and, while willing to use everything which will liberate a victim from intemperance, I am fearful that an absolute injustice is being perpetrated by the wholesale and persistent advertisement of this so-called cure. As I have said, I have yet to meet with a case in which it has been of any benefit whatever; nevertheless in the hope that my experience has been exceptional, I solicit an interview or correspondence with those who claim to have received benefit from or been cured by the cinchona rubrum. If it is a specific, I desire to know it; if it is not, I must be allowed to warn my patients against trusting in it.

That there was an urgent and constant demand for a speedy, simple and sure cure for intemperance, one which should, as it were, operate mechanically, relieving the patient of all responsibility and apprehension, pain and struggling, not only among those who are victims of the habit, but among their anxious and sympathizing friends and relatives, was well known to me.

I knew, too, that the human mind, even in a healthy state, is so ready to believe to be true that which the human heart most earnestly desires to be so, that many eager and credulous sufferers, who could ill afford it, would try the so-called unfailing specific before its clinical worth was established.

I did not feel it to be my duty, at that time, to accuse any one of charlatanism, but it did seem that a great and *grievous* wrong was being perpetrated, and that too, mainly upon a class who already have been robbed and depleted to absolute poverty. I

therefore wrote the above note, and, that it might be read by those particularly interested, published it in a newspaper.

It was not necessary for me to publish anything for the medical profession—every physician who had read even portions of the published letters, particularly in the *Tribune*, had decided in his own mind that the discoverer's premises were erroneous from the very outset. The assertion that alcohol is not absorbed, but passes directly to the brain *over* the nerves is enough to startle any physician, and it is not probable that any medical man of intelligence would peruse, at any length, an article containing the following: "Alcohol stimulates the nerve center—that is the brain—by passing *over* the nerves externally. If a man were to get drunk by the action of liquor upon the nervous system as other things taken into the stomach act, it would be about four hours before he felt it." Vide *Inter-Ocean* report. At page 16 in the pamphlet alluded to before, under the head "My Theory of Dipsomania," the same statement is again made.

The above is what the discoverer of the cinchona cure tells us. The facts are, that every physiologist from Magendie's time, who in 1809 made conclusive experiments, to the present, including Carpenter, Flint, Dalton, Kuss and others have demonstrated that absorption took place only through the blood vessels or lymphatics.

We have been aware that different soluble and volatile matters were absorbed with greater rapidity than others, always, however, through their proper channels, and while nerves do not absorb alcohol in the sense of conveying it from the stomach to the brain, they are profoundly affected by it through their blood supply. It is pretty generally admitted, too, by physiologists, that the reason why alcohol has this power, is because of its great affinity for albumen—nervous tissues being highly albuminous readily unite with the alcohol, to the extent *possibly* of changing the chemical composition of nerve tissue, though this is not positively known. The ease and rapidity with which chloroform, ether, camphor and ammonia are taken into the system is familiar to all. They are very volatile, but enter the circulation by the same process that a substance does whose absorption takes four hours. We are told, however, that alcohol, like electricity, goes over the sensitive nerves straight to the nerve cells, reaching them in a few minutes. The discussion for one moment of such a theory is useless, an absolute waste of time, an insult to the host of patient and scientific workers in physiology. If the author of this new theory will take the trouble to ligate all the blood-vessels running to the brain in one of the inferior animals, he will find that neither alcohol or any of the more easily absorbed substances will produce any effect whatever upon that organ. Absorption never takes place in, or through, or *over* the nerves.

The pathology of this gentleman is, if possible, more unique than his physiology:

"Drunkenness is a disease, a disease specifically caused. You will perceive after examining this drawing, that right in the center of these cells—this is a drawing that I made of the nerve cells seen within the cerebellum or smaller brain, through a microscope of three hundred and fifty diameter power—well, that black mark represents a "yellow fermentive substance; that is found only in the brain of one addicted to the use of alcoholic drinks. This substance is created and deposited there through the action of the alcohol taken into the system, upon the sugar contained within the blood. That which we term the appetite is nothing more than an expression of the nutritive wants of these fermentive products. I believe that I am thoroughly posted in regard to the nervous system, and I am satisfied that I can cure every case of dipsomania if the patient will but take the first dose of my remedy. The effect of cinchona rubra is a cumulative one, and its action is to destroy the power of this yellow ferment."

The facts in pathology are as follows: The pathological changes within the brain have not been well determined—the post-mortem appearance never revealing a constant deposit. The mode in which a fatal termination arrives, the congested blood-vessels, or, perhaps the ruptured artery, and also that remarkable tendency to excessive serosity, giving rise to what has been called a "wet brain," have been frequently observed. Rokitansky, Rindfleisch, Wagner, Green, Delafield, and many others, with every facility and appliance, have spent years in the careful examination of diseased tissue. Some of these observers have made 30,000 autopsies, and have examined the brain with instruments of the highest magnifying power in the possession of the most experienced microscopists in the world, and yet no constant and undeviating change has ever been found. In acute alcoholic poisoning where death takes place within from thirty minutes to several hours, there is frequently no change whatever in brain tissue. We find congestion of various organs and perhaps extravasations of blood in the brain and its membranes, but no change in nerve structure. Chronic alcoholic poisoning is however of a different nature. But even in this condition the patient may die of some other disease, or after a life-time of alcoholic excess die exhausted and comatose, and have in his brain and nervous system no change appreciable to the eye or microscope. A gentleman formerly under my care, and who was addicted not only to the alcohol but to the opium and chloral habit, fell dead on one of the streets of our city. The autopsy revealed the clot emptied from a ruptured blood-vessel but a minute microscopical examination showed no change or deposit in nerve cell. In other cases, however, we have found evidence of inflammation and hardening, particularly in what the eminent pathologist Virchow calls 'neuro-glia' (nerve cement) a kind of brain connective tissue. But it is not constant, and may be found in the brain of those not addicted to alcoholics.

Much has been published by this author to show that our knowledge of cinchona and its preparations has been greatly increased through his labors, but I am not aware that a single fact unknown to the profession, in regard to the therapeutical effect of this drug, has, up to this time, been advanced in any of his papers. The history of the drug is given in every work on Materia Medica, and its anti-periodic and tonic properties fully appreciated by all medical men of experience. That the red bark is as difficult to obtain as has been represented, and that the average pharmacist is so ignorant or dishonest as to make it impossible for the ordinary practitioner to obtain any of the cinchona preparations he may desire, I am not ready to believe. Indeed, all the statements made in regard to the scarcity of this drug, and in regard to a peculiar mode of manufacture, as well as its cumulative action, have been so put forward by the so called discoverer as to discourage any investigation on the part of the medical profession.

We have but few, if any, specifics, and medical men look with distrust and usually refuse to support any scheme *peculiar to, and in the interest of* any discoverer, or any theory based upon false, erroneous and antiquated hypotheses. The profession, however, is so ready for any scheme or plan, or medicine which will in any measure stop the increase of drunkenness, that notwithstanding the erroneous views promulgated at the outset by Dr. D'Unger, if experience has demonstrated that the drug can in any considerable degree do what he claimed for it, I have no doubt that full credit will be given to the doctor for his discovery, and its use very generally recommended by the majority of medical men. The profession demanded, however, a patient trial at the bed-side before deciding upon the merits of the preparation. At the end of one year, during which I have had the opportunity of observing the effect of the medicine in more than fifty cases, and also having had three analyses made by competent chemists, it appears proper that I should make known to the profession, and to many non-professional inquirers, anxious and interested however, the results of this new treatment.

The medicine is dispensed in small flasks and is seen to be a reddish muddy mixture having a distinct bitter taste and resembling in both taste and appearance a mixture of one part of common *tr. cinchonæ co.* with three parts of water. Sometimes I have seen a darker preparation, especially in the hands of an older drunkard, which is evidently a near approach to a fluid ext. of cinchona. This was true of a specimen analyzed by Prof. Haines to which I shall again refer. The original preparation of bark is manufactured, or was during the early part of the year, by Messrs. Gale and Blocki. The concentrating process is known to the discoverer only. The preparation from Messrs. Gale and Blocki is, of course, an excellent one, a clear, dark colored fluid extract; the concentrated tincture, "*six concentrations, to physicians* $10.00 *per pint*," prepared only by the discoverer, is a muddy mixture which alcohol will dissolve, showing that the concentrating process is performed by the addition of Lake Michigan water.

This expensive (?) mixture, some specimens of which contain as small quantity of bitter principle as one grain to the teaspoonful of water and alcohol is dispensed at from $5.00 to $10.00 per pint, with the following explicit

Directions for using Dr. D'Unger's Pure Cinchona Rubra.—The dose is a teaspoonful (shake the bottle well before each dose) once every three hours, the first dose always to be taken in the morning, before anything is eaten or drank. But if this dose of a teaspoonful causes any fullness or pain in the head, or any buzzing or ringing in the ears, the next dose must be a little less, say five drops; and so on, whenever there is any annoyance in the head, the dose must be made a little less.

Always remember, the dose must never be more than a teaspoonful, nor oftener than once every three hours. But if there is any desire for a drink of any kind of liquor between the doses, the party must moisten his or her mouth with a few drops of the tincture, but none of it (the tincture) must be swallowed except at the regular dose time.

There is no danger in using this remedy, but it must be taken as directed to insure success.

It is to be given only when the party is awake.

R. D'UNGER, M.D., *Discoverer*,
Chicago, Illinois, U. S. A.

The following is a correct copy of a contract given to one of Dr. D'Unger's patients. The original is in my posession:

Received of —— —— fifteen dollars, three-fifths of twenty-five dollars to be paid me for curing Mr. —— of dipsomania, the understanding being that if, at the end of ninety days, the said party is not cured, I am to return the fifteen dollars now received, and in case he is cured, the said Mr —— to pay me the remaining $10, I to furnish all medicine required.

R. D'UNGER, M.D.

And now what are the results? Exactly what one would expect after administering whisky and water with a small quantity of some bitter principle. I do not say that not a single person to whom this drug has been administered has stopped the use of liquors; but I do say that not one with whom I have come in contact has, and in many cases its use has rekindled an old desire which by resolution and education had been nearly conquered. I do not say that in every case following the administration of this nostrum, injury to the patient has come; but I do say that in every case which has fallen under my observation, this has been the result.

But what do the victims say. *Results:* CURES: this is what the people demand. "The proof of the pudding is in the eating." And so we come to the actual results. A mother writing in regard to her son, says:

DR. EARLE, Chicago, *Sir:*—You request to know how the cinchona rubra acted in ——— case. He is not able to answer, so I will write for him.

I think he knows very little about it, although he has taken the medicine, and thinks he has derived "no benefit from it." He came home sick with delirium.

Mr. ————, a cutter by occupation, makes the following statement:

I purchased a bottle of Dr. D'Unger's medicine, for which I paid him $5. He administered a dose to me before I left his office. The following night I felt the need of my usual stimulant, and drank the entire bottle of cinchona rubra. It seemed to do me the good that whisky usually did.

DR. EARLE—*Dear Sir:* Having heard that you wished me to give you my experience with the use of D'Unger's preventative and sure cure for drunkenness—cinchona rubra—it gives me great pleasure to furnish you with the following "bona fide" account of the effect it had on me:

In the first place, I wish to state that I acquired an appetite for whisky at an early age, having drank it more or less every day for the past sixteen years, and only abstaining from it when my money gave out, or when my credit was exhausted.

One afternoon (now about nine months ago) I met an old acquaintance —a thorough drunkard—J——C——, on the corner of Madison and Clark streets, in this city, both of us being without money, and with no earthly chance of getting a drink. He said to me: "Dave, I have a new scheme. I will go and see my old friend, Joe Medill (proprietor of the *Tribune*), he will give us a note to Dr. D'Unger, which will get us a bottle of his medicine, and that will cure us of drinking. I know several it has cured." He got the note, went to Dr. D'Unger's office, and received the medicine, with instructions as to how we should take it, with the assurance from him (Dr. D'Unger) that it would take away and destroy altogether the appetite for intoxicating drinks of every description. "I have cured," he said, "thousands of drunkards who were a great deal worse cases than you are." Well, John and myself took a walk by the lake front, for we had no home or boarding-house where we could go and take our medicine. After we had taken two or three doses, John asked me how I felt, and I told him it was burning the throat and stomach out of me. And in response to the same question from me, he said he thought it was making him drunk. Well, sir, instead of it destroying my appetite for drink, as D'Unger said it would, it certainly increased my appetite, for I imagined I would never get back from the lake-front to some of the hell holes I was in the habit of frequenting to get a drink of beer or whisky: and the next morning after taking D'Unger's cinchona, I found myself in a rum-hole on Dearborn street, rolling on the floor, covered with sawdust, with no hat, and without any shoes, both being stolen while I lay there beastly drunk. This is the CURE and the EFFECT that Dr. D'Unger's medicine produced on me.

I am now seven months old as a sober man in the Washingtonian Home, and I am happy to say that I am redeemed from drunkenness. The ONLY CURE that there is in this world, in my opinion, for a drunkard, is to abstain from all intoxicating drinks of every description, and if any man wants to become a sober and reformed man, no matter how great or degraded a drunkard he may have been, the Washingtonian Home is the place for him, for here he receives a moral, sober and religious education.

I am, sir, yours respectfully, D. B. WHELAN.

The following statement is made by the son of a State official in regard to the purchase of the medicine for a friend in a distant city, and its results:

I paid $10 for the first bottle, $10 for the second, $7.50 for the third, and the same for the fourth. My friend's appetite and inclinations are exactly the same as before taking the four bottles of medicine.

Mr. ———, a business man of the city, and the one who kindly furnished me his original contract from Dr. D'Unger, gives his experience as follows:

Consulted the doctor in April; was guaranteed a cure, and took a bottle of medicine. Have taken at least one-half dozen bottles since, and my appetite for whisky is the same as before I commenced, although the medicine was taken regularly according to directions.

Still another:

I have taken three bottles without deriving any benefit whatever, and upon ordering the fourth, requested the doctor to make it "double strength." This did me no good, and my appetite is the same as ever.

Dr. C. W. EARLE—*Dear Sir:* In trying to cure my appetite or desire for alcohol in all its forms, I was induced to consult Dr. D'Unger, and while under his treatment used two bottles of his cinchona. The only effect it had on me was an appetizer, and in fact I really believe my desire was stronger for alcoholic stimulants after using it. I gave it a fair trial—drank no liquor of any kind while using it—and in all candor must say it had no beneficial effect in my case. It may have proved a benefit in other cases—I only speak for myself. Respectfully, —— ——

CHICAGO, June 12, 1879.

DR. EARLE—*Dear Sir:* This morning I made a statement to you regarding the effects of the use of cinchona rubra upon my nervous system, but did not state fully how I was induced to take it. The facts are these, viz: Several gentlemen of my acquaintance who had been in the habit of drinking to excess, recommended it to me, and stated that since they had used it, they had no desire or appetite whatever for alcoholic drinks. This was also supported by Dr. D'Unger's statement that about three thousand persons who had been cured by his treatment of cinchona rubra, but since my sad experience lately in whisky drinking, I know of at least one of the gentlemen who recommended it to me who has returned to whisky, and so far as I know is drunk to-day. It may suit some temperaments, but certainly did him no good, and my late conduct proves it has done me none.

Respectfully, —— ——

CHICAGO, February 18, 1879.

DR. EARLE—*Dear Sir:* About a month ago I was feeling very nervous, and I thought it would be a good plan to try Dr. D'Unger's medicine. Accordingly I called on him, at his rooms in the Palmer House, and told him my condition—that the least little thing would frighten me. He told me the trouble was in the nerve center, and that no doubt his medicine would give me great relief. I told him that my financial condition was in a dilapidated condition, and that I could not afford to pay him $25 for his medicine. He asked me if I could raise $5, which I paid him and took a half-pint bottle of his remedy. I took it according to directions, and by the time I had finished the bottle I was in such a nervous condition that I had to stop work. It seemed to make me more nervous than I was before I commenced taking it. When the Doctor sold me the medicine for $5 he seemed to be doing an act of charity, as he assured me that each bottle cost him $7.50. I feel now that I made him a present of the money, and I have no hesitation in pronouncing him a first-class fraud, and a quack. His medicine is nothing more than a tonic, and I have often bought better ones for twenty-five cents.

Respectfully, —— ——

P. S.—Had not the *Tribune* backed this man up I never would have called on him at all.

Chicago, June 13, 1879.

Dr. Earle—*Dear Sir:* You will here find my limited experience in the use of Dr. D'Unger's cinchona cure. I commenced taking it about three months ago, and at first it made my head feel very badly, about the same as an over-dose of quinine would; making my head feel enlarged; and causing my ears to ring. Immediately after taking it I would feel also inclined to take a drink to relieve the feeling. It had the same effect that a drink of whisky would have—that is a desire to follow it with another drink. I complained of this to my wife, but she thought it only an excuse on my part to get rid of taking the medicine, and she concluded she would try it, but with the same result, excepting she did not desire to drink after it.

I then made up my mind that there was alcohol in it. I poured some of it in a dish, then set fire to it, and found it to burn as freely as any spirit. I am fully satisfied it could never cure me from drinking, and, as far as I am personally concerned, I consider it an unmitigated humbug, and will have none of it. I prefer the Washingtonian Home. I have part of a bottle left, which is at the disposal of any unfortunate who may wish to try it, and not be obliged to pay the exhorbitant $15 for a pint of his "infallible cure." After my wife trying it and experiencing the feeling which she did, she was perfectly willing for me to discontinue its use.

You are at liberty to use this as you may choose.

### RESULTS FROM ABROAD.

Dr. C. W. Earle—*Dear Sir:* In response to your request, I will state that I have been a resident of Minneapolis until recently; that I am acquainted with Dr. D'Unger, both personally and by reputation; that I was Grand Worthy Chief Templar of the Order of Good Templars for two years; that during said time I was aware that Dr. D'Unger claimed a sovereign cure for intemperance (through the newspapers) in cinchona rubra, but with the fact in view that I was gathering all the facts possible for my temperance work, I have never known of one single authentic instance, where cinchona effected a permanent cure Respectfully,

A. M. Hutchinson.

Minneapolis, Minn., Jan. 9, 1879.

Chas. W. Earle—*Dear Doctor:* I have delayed answering yours of the 3d inst., that I might see other physicians concerning the D'Unger treatment. I can only hear of two cases that have been treated here. One with no benefit and the other (colored) seemingly improved. Should I hear anything that would be of use to you, will be glad to send the information.

Yours very truly, F. A. Dunsmore, m.d.

Minneapolis, Minn., Dec. 28, 1879.

C. W. Earle, m.d.—*Dear Doctor:* Yours of the 20th inst. is now before me and since its reception I have had opportunity of asking a number of our best physicians for their results with D'Unger's cure by cinchona bark. No one reports success but majority have not made experiment, their excuse being their knowledge of the doctor. I have prescribed but with no knowlege of result, which I would certainly have received had there been any success. Very truly yours, F. A. Dunsmore, m.d.

C. W. Earle—*Dear Sir:* In reply to your inquiry concerning Dr. D'Unger and his pretended cure for drunkenness, permit me to say that I am now and have been for several years a resident of Minneapolis: that I am personally and well acquainted with Dr. D'Unger, and while I have been constantly engaged in the temperance work in that city, I do not know of one individual, nor have I heard of one who was cured, or who claimed to have been cured through the instrumentality of cinchona rubra. I once induced a man, who had been a hard drinker, to partake of the cinchona rubra, with a view of testing its merits, but the result was very unsatisfactory, the partaker complaining of an almost insatiable thirst for drink for several hours after taking the medicine. Very truly yours,

J. C. Erwin,
Grand Sec'y Good Templars, Minn.

Dr. R. J. Patterson, formerly Professor of Medical jurisprudence, Chicago Medical College, and at present the proprietor and superintendent of Bellevue Place, a private institute for the treatment and cure of insane females, Batavia, Ill., furnishes me the history of a case which illustrates not only the usual (as far as I can learn) result, but the cost of making the trial:

A lady, the wife of an eminently respectable business gentleman, residing in a neighboring city, had become addicted to alcoholics—she was possibly a victim to the morphine habit—and had been committed to an asylum in the city several times but to no purpose. She seemed incurable. At last she came to Chicago, and was guaranteed a cure by Dr. D'Unger. One hundred dollars was paid for the guaranteed cure. While she was taking the medicine and under observation, it seemed to be all the stimulant necessary, but a few days after her return home she was again under the influence of alcoholics, and had recently been an inmate of Dr. Patterson's retreat.

RESULTS AT THE NATIONAL HOME FOR DISABLED SOLDIERS.

A short time after I published my card in the *Tribune*, the following was displayed as an advertisement:

DRUNKENNESS CURED—FACTS VS. FOLLY.—"NATIONAL HOME FOR DISABLED VOLUNTEER SOLDIERS, DAYTON, O., Jan. 6, 1879.—*Dr. D'Unger, Chicago:* After a fair trial of three months in two cases, seemingly incurable, I am glad to say that the result has been all that you could claim and all the most sanguine could hope for. Respectfully, E. F. BROWN, Governor."

A few days since I addressed a letter to Col. Brown, asking him if, after a continued trial of the remedy, he could still recommend it. His answer is probably explicit enough for even the discoverer of the so-called specific for drunkenness:

DAYTON, OHIO, Dec. 12, 1879.

DR. CHAS. W. EARLE, 37 Park ave., Chicago, Ill.—*Dear Sir:* Your favor of yesterday is received, and contents noted. I gave the cinchona a fair trial, and found it not better than any other advertised nostrum for the cure of intemperance. But it is proper to say I was not more sanguine of success when I wrote Dr. D'Unger in regard to the purchase of a quantity of the remedy than the facts seemed to warrant. He claimed for it that after three months' use the desire for all stimulants would pass away, and a cure be wrought, and in the two instances to which, doubtless, you refer, the men did keep sober that long. Thus far the remedy, or the men's resolution—it is hard to tell which—was efficacious, and thus far only was I sanguine. I do not hesitate now now to pronounce it a humbug.

Very respectfully yours, E. F. BROWN, Governor.

Although the uniformity of these results may be somewhat monotonous and lengthy, and the reader may long for the record of at least one reformation, it is impossible for us to give it. But as no record of so-called cures would be at all complete without slight reference to the published cures from among the inmates of the Washingtonian Home, I must be pardoned for giving the completed history of those four remarkable (?) cures, already referred to in the opening page of this paper and found at page fifteen in the doctor's treatise on dipsomania.

He says: "at the Chicago Washingtonian Home, four of the patients of the worst description * * * have been completely cured by the use of my cinchona rubra. These patients can be seen personally by parties interested." The only truth in the entire sentence is that they can be seen. I have seen them; all are or have been drunk. The history of case No. I. has been partly given in one of the letters which appear above. A few days ago a commercial traveler called at the young gentleman's residence, in the central part of our State, and found that the day before, the young man while under the effect of liquor had demanded money of his father, and on being refused, the reformed man (?) "who may be seen by parties interested," proceeded to chastise his paternal relative with an ax handle.

CASE II. Was a Southern gentleman of position and education. He has made repeated efforts at reformation, but to no effect. At last a prominent business gentleman of this city became interested in him and at the commencement of the cinchona craze paid $25.00 for the reformation of the case we are considering. The patient professed to be greatly invigorated by the use of the medicine, and believed now his reformation complete. While this gentleman was taking the cinchona of Dr. D'Unger I asked him to compare it with an aqueous fluid extract of red bark made by Sargent & Co., of this city for my use. The preparation which this firm had made for me contained one pound of bark to the pint, and was absolutely free from alcohol. It was an exceedingly strong preparation, and would do for a patient all that Peruvian bark is capable of doing. He tasted this aqueous ext., and immediately said to me, "you haven't got it. It's not the thing." I had given him a dose of cinchona containing at least twenty times as much of the alkaloid as he was taking in the D'Unger preparation, but "it wasn't the thing." It did not contain from two to twenty-four per cent. of alcohol, which our reformer's tincture does, and hence these tears. After taking several bottles of this nostrum this gentleman engaged in business as a commercial traveler, I think, but soon returned to the city drunk. He entered Mercy hospital for a time but after leaving that institution again relapsed, and was reduced to a very pitiable condition. Upon my personal solicitation he was again admitted to the Home, and after a season resumed business. He is now in a distant State, and his future as regards drinking, is exactly what he makes it. He is a strong, robust man and can abstain from drink if he wills.

CASE III. Is a newspaper man, an editorial writer, one of those men who can paint with words; a most able, accomplished and affable gentleman. He took the D'Unger mixture to remove the appetite. While taking it he was engaged in business, and at length determined to visit the East, his former home. He returned to this city drunk, his money squandered, his family discouraged. Repeated efforts were made by several to even sober him up without admitting him to the Washingtonian Home, but without success; and notwithstanding a large amount of abuse had been directed to the Home and its physician, and encomiums and peans of praise showered down on "the greatest benefactor of his race," as the discoverer of this cure has been called; notwithstanding all this, the man was again admitted and has again gone out and is at his accustomed work.

CASE IV. This man was the last one to hear from—the only one in the list in whom "the greatest discovery of the age" had not been demonstrated a failure. Three weeks ago his wife came to the institution referred to above and stated that he was drinking, and his position lost. He is now in the Home, where, to use the words of my friend D'Unger, "he may be seen by parties interested."

FROM THE MEDICAL JOURNALS.

The Edinburgh (Scotland) *Medical Journal* says of the cinchona, or Peruvian bark, remedy for drunkenness:

"Having been pressed by the friends of an unfortunate drunkard to try this new remedy, we administered it exactly as directed. We found the red bark a good tonic—this and nothing more. Our patient visited us some time after his convalescence, with a powerful odor of alcohol about him, and we ascertained from his distressed friends that he is pretty much what he was. We are inclined to think the whole thing another American dodge—and a very clever one. The moral is a sad one."

I beg leave to say to the editor of this journal that although this is an "American dodge," the medical profession is not responsible for it. The author of this humbug is not accredited by any medical society or association in our broad land.

The *Medical and Surgical Reporter*, of October 18th, says:

"We duly noted this vaunted cure on its announcement; we now chronicle its worthlessness. Dr. A. P. Hayne, superintendent of the Home of the Inebriates, San Francisco, writes thus of it, after full trial, in the *Western Lancet:*

"In no single instance have I found it to possess the slightest power in disgusting the patient with liquor or in any way diminishing his or her appetite or craving for intoxicating drinks. On the contrary, they have all freely confessed to me that the anticipation and the pleasure of a drink was as strong as ever, In no case have I seen whisky refused when offered and in the majority it has been asked for. I know of an instance, in private practice, in which a person has stayed at home and taken the remedy all day, for several days in succession, and gone out in the evening only to be brought home thoroughly intoxicated.

ANALYSIS.

DR. EARLE—*My Dear Doctor:* The following is the result of the examination of the two specimens you left at the college:

The darkest colored liquid in the salt-mouth bottle contained of

Alcohol.................................16 per cent.

Water.................................85.19 per cent.

Total solid matter about six grains.

The liquid in the second bottle contained of

Alcohol................................. 2.7 per cent.

Water.................................98. per cent.

Total solid matter about four grains.

The percentage is by volume, and as water and alcohol mixed, contracts in volume the apparent discrepancy in the figures is explained.

The precipitates found in each specimen were, undoubtedly, caused by the addition of water to an alcoholic tincture as they were soluble in alcohol.

The amount of solid matter was so small that I did not attempt to ascertain the alkaloids present. Yours truly,

P. S. HAYES,

Prof. Chemistry, Woman's Medical College.

CHICAGO, Dec. 22, 1879.

PROF. C. W. EARLE, M.D.—*Dear Sir:* The sample of Dr. D'Unger's cinchona rubra preparation, which you placed in my hands for examination has been analyzed, and found to contain twenty-four per cent. of absolute alcohol. Yours truly, WALTER S. HAINES, M.D.,

Prof. of Chemistry and Toxicology,

Rush Medical College.

So much then for a specific which is persistently advertised as curing *every case* of drunkenness. The scheme is an exceedingly ingenious one. The patient, supposing he is supplied with a wonderfully powerful drug, possessing great tonic and anti-periodic properties, is really given an amount of alcohol varying from two to twenty-four per cent., the remainder of the nostrum being water. In this we have a very small amount of a bitter principle, presumably Peruvian bark. The first dose is *always to be taken* in the morning, before anything is eaten or drank—see printed instructions. This first dose of alcohol and water, with a little bitter, before anything is eaten or drank, supplies the usual first drink which is so urgently demanded by one addicted to alcoholics. Then a teaspoonful every three hours, and if there is a desire for a drink of any kind of liquor between the doses, the party must moisten his or her mouth with a few drops of the tincture—twenty-four per cent. alcohol. Now, it is a well known fact that a man who has once acquired a taste for alcoholics, but by resolution and education of his moral sense has been enabled to reform, can not, with safety, taste a drop of any kind of liquor. In many of these cases a desire for liquor remains, and with the hope that this longing may be destroyed, many have been induced to take this prescription, and in *every case*, as far as my observation extends, they have again fallen. But I cannot prolong my paper. Much remains which might be said. *Every man*, with one exception, as far as I can learn (if I am mistaken I shall be glad to be corrected), whose reformation was published in the *Tribune*, is or has been drinking. I saw one in the County Hospital a few days since; he remained sober for three months, while taking the cure, and then drank ordinary whisky for nine months; and yet this man assured me that he was reformed; he had no taste for liquor, and yet he had been under its influence for nine months. Another man who formerly drank very largely of beer and who was occasionally unkind to his family, after a course of D'Unger tincture, had selected whisky as his beverage. He drank less whisky than beer, but beat his wife more. It would seem from this that beer is the better family drink. Others are taking the Peruvian tincture during the forenoon while business is active, changing to a different liquor after business hours.

Before concluding it is perhaps proper for me to say that I understand well that an *expose* of this humbug may be followed by a deluge of abuse and criticisms, which is not particularly pleasant. If, however, friends will bear in mind that every letter of abuse which has, up to this time, appeared, has come directly or indirectly from those who have been discharged, or have had friends discharged, from the Washingtonian Home, either on account of violation of some rule of discipline, or because they were not proper persons for us to retain, the animus of these letters will be seen. The committee on admission and discharge in that institution cannot, and does not, expect to do its full duty without some very severe criticism.

CONCLUSIONS.

1. A chemical examination of the D'Unger preparation of so-called concentrated cinchona rubra shows it to be a diluted mixture of fluid extract of cinchona with water.

2. The amount of absolute alcohol is from two to twenty-four per cent.

3. The amount of bitter principle is as small, in some specimens as one grain to the drachm.

4. Engaged in a hospital practice where I have prescribed for nearly four hundred cases of alcoholism during the year, in addition to a private practice in which I see, perhaps, as many of these cases as the average physician, I have yet to see the first reformation from its use.

5. *In not a single case* has the use of this preparation disgusted the patient with the taste of alcohol.

6. The taste for stimulants in many cases remains long after a reformation is complete. Indeed, it is never lost in some, and a constant fight goes on between a desire for some form of stimulants and duty made plain by the education of the moral sense, to abstain from them. Numbers of these men, encouraged by the repeated assertion that this taste could be certainly and safely destroyed, have taken this medicine. In *every case* it has been the direct and only cause of these patients returning to their former sad and terrible habits. It has caused the downfall of every one who has come under my observation belonging to this class who has dared to touch it.

7. From a careful investigation of all the facts in my posession, I desire to place on record that it is my belief that this cinchona treatment has made more drunkards during the past year in this city than any one saloon.

